# Drug Signature-based Finding of Additional Clinical Use of LC28-0126 for Neutrophilic Bronchial Asthma

**DOI:** 10.1038/srep17784

**Published:** 2015-12-02

**Authors:** Eunji Shin, Yong Chul Lee, So Ri Kim, Soon Ha Kim, Joonghoon Park

**Affiliations:** 1 LG Life Sciences R&D Park, Daejeon, 305-380, Republic of Korea; 2 Department of Internal Medicine, Research Center for Pulmonary Disorder, Chonbuk National University Medical School, Research Institute of Clinical Medicine of Chonbuk National University-Biomedical Research Institute of Chonbuk National University Hospital, Jeonju, 561-180, Republic of Korea

## Abstract

In recent decades, global pharmaceutical companies have suffered from an R&D innovation gap between the increased cost of a new drug’s development and the decreased number of approvals. Drug repositioning offers another opportunity to fill the gap because the approved drugs have a known safety profile for human use, allowing for a reduction of the overall cost of drug development by eliminating rigorous safety assessment. In this study, we compared the transcriptional profile of LC28-0126, an investigational drug for acute myocardial infarction (MI) at clinical trial, obtained from healthy male subjects with molecular activity profiles in the Connectivity Map. We identified dyphilline, an FDA-approved drug for bronchial asthma, as a top ranked connection with LC28-0126. Subsequently, we demonstrated that LC28-0126 effectively ameliorates the pathophysiology of neutrophilic bronchial asthma in OVA_LPS_-OVA mice accompanied with a reduction of inflammatory cell counts in the bronchoalveolar lavage fluid (BALF), inhibition of the release of proinflammatory cytokines, relief of airway hyperactivity, and improvement of histopathological changes in the lung. Taken together, we suggest that LC28-0126 could be a potential therapeutic for bronchial asthma. In addition, this study demonstrated the potential general utility of computational drug repositioning using clinical profiles of the investigational drug.

Most pharmaceutical companies have oriented toward target-based drug discovery in a challenging effort to discover novel targets. It is generally followed by costly and risky processes to bring a new drug to market^1^. Compared to the early 1990s, the developmental cost for a drug has risen, and the consolidated regulatory hurdles for adherence to safety requirements coupled with costly, novel technologies drive this rise. Although the developmental cost of a new drug sharply increases, the number of new drugs approved stays steady. To mitigate this innovation gap, many pharmaceutical companies practice open innovation to get candidates into late-stage development, use merge and acquisitions to expand business opportunities, or address targeted patient populations with specialty products; however, the revenue from these innovative activities turns out to be unsatisfactory^2^.

Drug repositioning offers another opportunity to fill the innovation gap. The approved drugs have a known safety profile for human use, and the application of these drugs to new therapeutic indications can reduce the overall cost of drug development by almost 40% by eliminating the rigorous safety assessments^3^. As exemplified by sildenafil, which was originally tested for angina but approved for erectile dysfunction, or by thalidomide, which has been withdrawn as a sedative but re-marketed for multiple myeloma and leprosy, unintended adverse events led to new indications^4^. The recent advancement of bioinformatics allows rational approaches on drug repositioning during the developmental phase or after approval. Computational drug repositioning can be categorized into disease-based and drug-based approaches^5^. In the disease-based approach, a molecular network of disease and/or side effect similarities can guide the discovery of a new indication of the existing drugs^6^,^7^. Topiramate, an anticonvulsant, was identified as a potential therapeutics for inflammatory bowel disease through the disease-based approach^8^. In drug-based repositioning, chemical similarities and/or the mode of molecular docking to a novel target can enable a discovery of alternative indications of the existing drugs by using a quantitative structure-activity relationship (QSAR) or virtual screening^9^,^10^. However, even though the similarity of the structures and/or the docking mode of drugs can aid in drug repositioning, they do not always trigger the same biological response. Compared to these conventional drug-based approaches, the similarities of the biological responses of drugs can be measured by genome-wide transcriptional profiling; thus, it can provide another opportunity for drug-based repositioning.

LC28-0126 is a novel class of mitochondria-targeted reactive oxygen species (ROS)/reactive nitrogen species (RNS) scavenger that was discovered through the function-based approach to cellular necrosis and inflammation^11^,^12^. Necrosis is tremendously important in the pathogenesis of MI, stroke, and neurodegenerative diseases. LC28-0126 effectively ameliorates the pathophysiology against oxidative stress-induced damage through the inhibition of high-mobility-group-box 1 (HMGB1) release, inhibition of mitochondrial NADPH oxidase complex activity, inhibition of mitochondrial calcium uniporter activity, inhibition of nuclear localization of NF-κB, inhibition of mitochondrial permeability transition pore opening, and regulation of regulatory T cells^13^,^14^,^15^. The preservation of the mitochondrial function and/or scavenging of the mitochondrial ROS are especially essential to limit cardiomyopathy from ischemic-reperfusion injury. More than 1 million cases of MI occur in the United States every year. Patients with MI typically exhibited a large area (~70%) of infarction of heart (www.nhlbi.nih.gov), causing death from heart attack. Several clinical trials performed in patients with MI have failed, as they did not demonstrate substantial reduction in size of the infarcted area, which is decisively influenced by the degree of necrosis of cardiomyocytes. Therefore, LC28-0126 is under active development for evaluation of the efficacy, safety, and pharmacokinetics by intravenous single injection immediately before percutaneous coronary intervention in ST-segment-elevation myocardial infarction patients at phase 2 clinical trial (ClinicalTrial.gov Identifier: NCT02070471).

In this study, we aimed to discover a new clinical indication of LC28-0126 through drug-based computational drug repositioning. To this end, we analyzed cytokine profiles in healthy subjects to evaluate any undesired inflammatory reactions by LC28-0126 in a non-pathological condition. We then compared the transcriptional profile of LC28-0126 obtained from healthy subjects with the molecular activity profiles of 1,309 compounds in the Connectivity Map^16^,^17^ to identify a top-ranked connection of possible clinical use. Subsequently, we demonstrated that LC28-0126 effectively ameliorates the pathophysiology of neutrophilic bronchial asthma in OVA_LPS_-OVA-induced asthmatic mice without significant toxicological effects.

## Results

### Cytokine profile in healthy subjects by LC28-0126

Cytokine profiling revealed that LC28-0126 did not change the expression levels of cytokines 6 hrs after single infusion in healthy subjects (Table 1, Supplementary Table S1). Most of the inflammatory and Th1/Th2/Th17 cytokines were measured at detection limits or below. Levels of TNF at 10 mg per person and IL-2 at 1 mg per person of LC28-0126 were significantly increased after infusion (*p* < 0.05), but they were within normal range. These results indicate that at least single infusion of LC28-0126 would not induce pathological inflammatory response in healthy subjects.

### Transcriptional response by LC28-0126 in healthy subjects

ComparativeMarkerSelection revealed that 15, 51, 12, and 15 genes were differentially expressed 6 hrs after LC28-0126 infusion at 1, 3, 10, and 25 mg per person, respectively. Out of 63 probes, 49 genes were identified as differentially expressed by LC28-0126 in healthy subjects (see Methods for DEG criteria; Supplementary Table S2). Among them, phospholipase A2, group IVA (*PLA2G4A*), adrenergic, beta-1, receptor (*ADRB1*), and thrombospondin receptor (*CD36*) were upregulated throughout entire dose levels of LC28-0126. In contrast, pyruvate dehydrogenase kinase, isozyme 4 (*PDK4*) was downregulated by LC28-0126 (Table 2). Functional annotations indicate that these genes have clinical relevance in cardioprotection and/or inflammatory response. For example, *PLA2G4A* participates in membrane phospholipid metabolism, and upregulated *PLA2G4A* results in the production of cardioprotective eicosanoids like prostaglandin E2 (PGE2)^18^,^19^,^20^. MI is accompanied by a release of endogenous catecholamines for β-adrenergic stimulation, resulting in myocardial damage^21^. Early-phase ischemic preconditioning preserves myocardial function by attenuating mitochondrial matrix calcium overload, apoptosis, and additional ROS production at reperfusion^22^. Interestingly, the noradrenaline-depleted hearts were significantly protected when conditioned with isoproterenol, a non-selective beta-adrenergic (*ADRB1* and *ADRB2*) agonist for the treatment of bradycardia and heart block, before global ischemia^23^. This protective effect was eliminated by blocking the ADRB1 during ischemic preconditioning^24^. These results imply that *ADRB1* may mediate both myocardial protection and damage during ischemia. C*D36* is a membranous long-chain fatty acid (FA) translocase that plays an important role in cardioprotection against ischemic injury through supply of a major energy substrate in the heart^25^. Loss of function of *CD36* is associated with reduced tolerance to myocardial ischemic/reperfusion injury in *CD36* null mice^26^ as well as a defect in the uptake of long-chain FA in the heart of patients harboring *CD36* mutations^27^. Pyruvate dehydrogenase complex (PDC) generates ATP and FA in mitochondria through oxidative decarboxylation of pyruvate derived from glycolysis^28^. PDC is activated by pyruvate dehydrogenase phosphate phosphatases^29^ and inactivated by pyruvate dehydrogenase kinases (PDKs)^30^. *PDK4* is a skeletal- and heart-muscle-specific PDK^31^, and its expression is associated with the energy homeostasis and acute MI^28^,^32^. Taken together, the transcriptional response of LC28-0126 could be obtained from the healthy subjects, and it is consistent with the clinical potential of LC28-0126 for necrotic cell death accompanied with inflammation in MI^11^,^13^.

### Statistically significant connections of LC28-0126 to reference drug signatures

**Figure 1:**
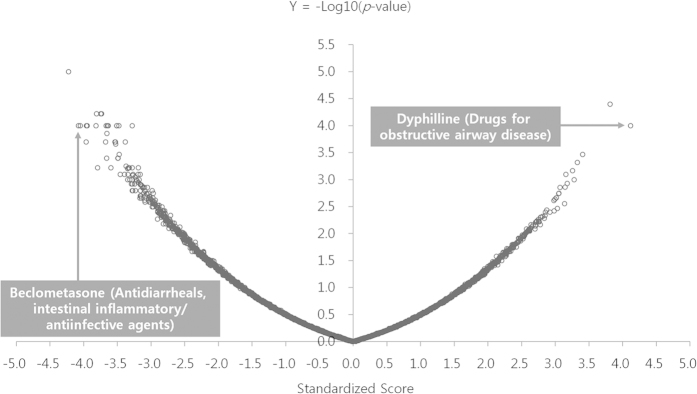
Statistically significant connections between LC28-0126 and Lufyllin. Volcano plot displays the LC28-0126 signature compared with a reference collection of gene expression signatures in the Connectivity Map. The x-axis is for the standardized connectivity score, while the y-axis is for –log_10_ (*p*-value). Dyphillin, an active ingredient of Lufyllin, was top ranked to have comparable molecular activity of LC28-0126 (*p* = 0.0001, standardized score = 4.12), and Beclometasone was top ranked as an opposite signature to LC28-0126 (*p* = 0.00001, standardized score = −4.22).

Out of 49DEGs by LC28-0126 treatment, 24 DEGs which were matched with gene ID in the Connectivity Map instance were used as a query gene signature. The sscMap revealed that LC28-0126 had 8 similar and 57 opposite drug signatures in the Connectivity Map (Fig. 1, Supplementary Table S3; *p* < 0.00126, standardized score >|3.1|). The anatomical therapeutic chemical (ATC) classification system indicates that the connected drugs are in use in various ranges of indications including obstructive airway disease, nervous system, urologicals, cardiac therapy, vasoprotectives, cough and cold preparations, and intestinal anti-inflammatory agents (Table 3). Among the similar drug signatures, dyphillin, an active ingredient of Lufyllin, was top ranked to have comparable molecular activity of LC28-0126 (*p* = 0.0001, standardized score = 4.12). Lufyllin was approved for relief of acute bronchial asthma and for relief of reversible bronchospasm associated with chronic bronchitis and emphysema by the US Food and Drug Administration in 1976 (http://dailymed.nlm.nih.gov). Dyphillin is a derivative of xanthine in a group of alkaloids, and it is supposed to act as both a competitive nonselective phosphodiesterase inhibitor to reduce inflammation and innate immunity and a nonselective adenosine receptor antagonist^33^. Beclometasone was top ranked as an opposite signature to LC28-0126 (*p* = 0.00001, standardized score = −4.22). Beclometasone is a potent glucocorticosteroid and has been prescribed to reduce swelling in the airways caused by severe asthma (http://www.nlm.nih.gov/medlineplus). However, glucocorticosteroids have pleiotropic effects including immunologic, metabolic, developmental, and cognitive activities as well as body fluid homeostasis^34^; therefore, it raises concerns over non-specific activity on asthma.

### Anti-asthmatic effect of LC28-0126 in OVA_LPS_-OVA mice

**Figure 2:**
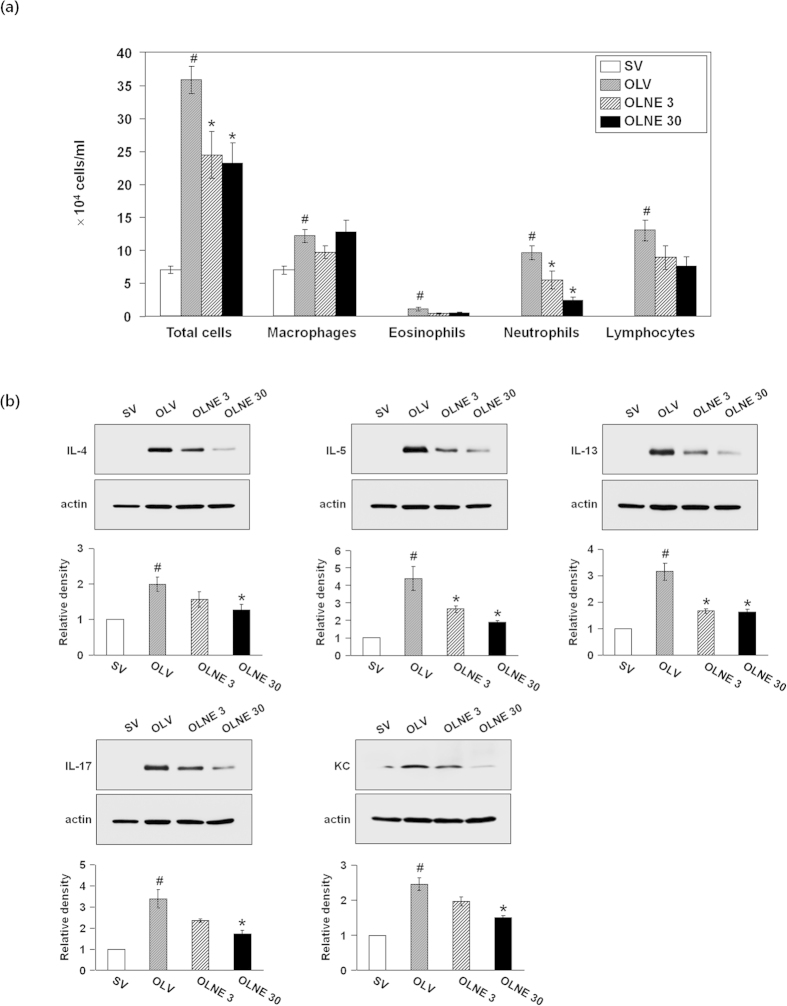
Effects of LC28-0126 on differential cell counts and protein levels of inflammatory cytokines in OVA_LPS_-OVA mice. All parameters were measured at 48 hrs after the last challenge in saline-sensitized and challenged mice administered with drug vehicle (SV), OVA_LPS_-OVA mice with drug vehicle (OLV), OVA_LPS_-OVA mice 3 mg/kg of LC28-0126 (OLNE3), or OVA_LPS_-OVA mice with 30 mg/kg of LC28-0126 (OLNE30) (**a**) Differential cell counts in BAL fluids in LC28-0126-treated OVA_LPS_-OVA mice. Bars represent mean ± SEM from 7 mice per group. (**b**) Expression of inflammatory mediators in LC28-0126-treated OVA_LPS_-OVA mice. Bars represent mean ± SEM from 5 mice per group. ^#^*p* < 0.05 vs. SV or Control; *p < 0.05 vs. OLV.

**Figure 3:**
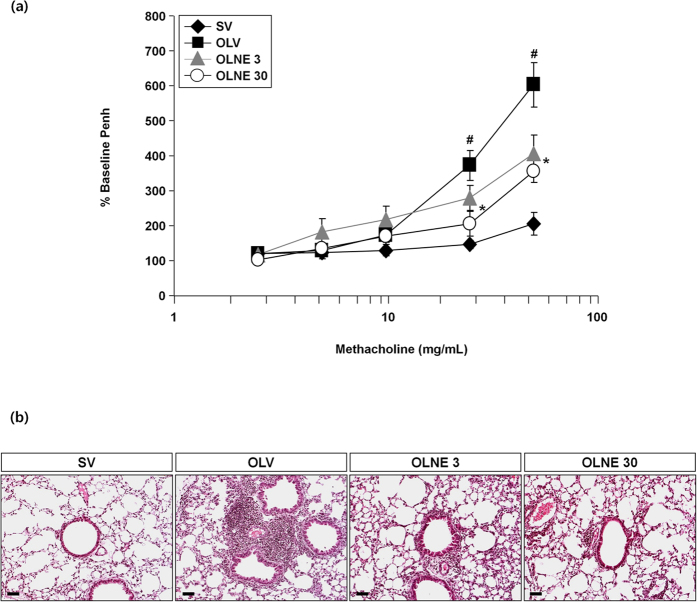
Effects of LC28-0126 on airway responsiveness and histological changes in OVA_LPS_-OVA mice. (**a**) Airway responsiveness in LC28-0126-treated OVA_LPS_-OVA mice. Bars represent mean ± SEM from 8 mice per group. #*p* < 0.05 vs. SV or Control; *p < 0.05 vs. OLV. (**b**) Histopathological changes in airway in LC28-0126-treated OVA_LPS_-OVA mice. Scale bars indicate scale of 50 μm.

In OVA_LPS_-OVA mice, the numbers of total cells, macrophages, eosinophils, neutrophils, and lymphocytes were significantly increased in the BALF compared to saline-sensitized control mice (*p* < 0.05). LC28-0126 at 3 mg/kg and 30 mg/kg bodyweight significantly reduced the numbers of total cells and neutrophils in the fluid in a dose-dependent manner (*p* < 0.05) (Fig. 2a).The expression levels of proinflammatory cytokines were significantly increased in the lung tissues of OVA_LPS_-OVA mice compared to saline-sensitized control mice (*p* < 0.05), and they were significantly decreased by LC28-0126 in a dose-dependent manner (Fig. 2b). The airway of OVA_LPS_-OVA mice was hyperactive to methacholine at ≥30 mg/mL in comparison with saline-sensitized control mice (*p* < 0.05). LC28-0126 ameliorated the airway response to the methacholin dose proportionally, and Penh values were significantly reduced at ≥30 mg/mL of methacholin (*p* < 0.05; Fig. 3a). Histopathological examination revealed thatthere was a severe inflammatory response in the lung of OVA_LPS_-OVA mice, characterized by numerous inflammatory cells infiltrated into the bronchioles and perivascular regions and goblet cell hyperplasia, and it was markedly relieved by LC28-0126 treatment (Fig. 3b). A 28-day intravenous repeat dose toxicity study in ICR mice with LC28-0126 at 40 or 80 mg/kg/day revealed that LC28-0126 is tolerable even at 80 mg/kg/day. No specific organ toxicity was observed, including hematology and lung. This verifies the anti-asthmatic effect of LC28-0126 and rules out a generic cytostatic or an unintended effect (Supplementary Table S4).

## Discussion

In an effort to extend the clinical use of LC28-0126, we applied the drug-based computational drug repositioning by means of the similarity comparison of the molecular activity of LC28-0126 obtained from the Phase 1 clinical trial to the molecular activity profiles of reference compounds in the Connectivity Map.

In this study, we observed the highest number of differentially expressed genes at 3 mg/person of LC28-0126, an intermediate dose level. Compared to this, low and high dose levels of LC28-0126 have little effect on differential expression of genes. This dose-independent transcriptional response could be explained by the ROS scavenging property of LC28-0126. ROS are one of the important intracellular signaling molecules for differentiation, growth, cell death, and senescence. Paradoxically, ROS serve as both an initiator and a terminator of the ROS-mediated cellular reactions^35^. This hormetic feature of ROS elicits the bell-shaped dose-response by antioxidants^36^,^37^. Therefore, these results may reflect the dichotomy of ROS-mediated transcriptional activity, and 3 mg/person of LC28-0126 would be the optimal dose level to protect from injury to the post-ischemic heart, at least in aspect of transcriptional regulation.

To our surprise, the transcriptional signature of LC28-0126 in healthy subjects is composed of cardioprotective genes, such as *PLA2G4A*, *ADRB1*, *CD36*, and *PDK4*, working against oxidative stress. The enriched biological ontology is consistent with our previous finding in rats^13^, even though the signaling molecules are not exactly the same between rat cardiomyocytes and human PBMCs. It is largely recognized that different organs, tissues or cells might have distinct patterns of gene expression; furthermore, cells in different pathological conditions might respond differently to the same stimuli^38^. However, several lines of empirical evidence demonstrate that the biological response could be conserved across diverse cells from different origins of tissues or different pathological conditions^8^,^16^. To identify the common mechanism of action of LC28-0126 in rat cardiomyocytes and human PMBCs, we made use of non-parametric statistics along with a genome-wide, ranked list of genes rather than individual profiles of fold-change. Through this approach, we could avoid the bias from the experimental setting and identify the common molecular activity. It is also noteworthy that ROS are definitive substrates of LC28-0126. ROS play an important role in the regulation of gene expression. ROS activate transcription factors like NF-κB, which bind to promoter regions of some ROS-dependent genes, including prostaglandin-endoperoxide synthase 2 (*PTGS2*) and homeobox A1 (*HOXA1*), for activation^39^. Although it is largely unknown what makes certain genes sensitive to ROS, the consistent molecular signature of LC28-0126 in humans and rats could be explained by its intrinsic property as an antioxidant. In essence, LC28-0126 would induce similar effects both in non-pathological and pathological conditions, and the degree of the effects could be differentiated depending on the condition.

The signature of LC28-0126 had a significant connection with dyphilline for bronchial asthma. Asthma is an inflammatory disorder of the airway characterized by the infiltration of inflammatory cells and incitement of Th2-type cytokines in the lung, airway hyper-responsiveness, reversible airflow obstruction, and shortness of breath^40^. According to WHO estimates, as many as 300 million people suffer from asthma worldwide, and children are mostly affected. Asthma has been treated with bronchodilators, systemic xanthines, and corticosteroids. By the mid 2000s, newly defined immunological pathways in asthma opened up new, emerging therapeutics, including leukotriene modifiers and anti-immunoglobulin E antibodies; however, most therapeutics are not working in clinical practice^41^. Asthma has necrotic phenotype, including inflammation, excessive generation of ROS, ATP reduction, and mitochondrial swelling and dysfunction. Because of these pathophysiological features, several antioxidants have been investigated to reduce and/or prevent asthma; however, most of the antioxidants are not chosen for clinical application for asthma due to limited access to the ROS generating mitochondria.

Classically, asthma is a chronic airway disease with origins in childhood, and it is related to allergies, dysregulated Th2 inflammatory response, and eosinophils. Therefore, it has been best treated by targeting inflammation. However, several recent studies are now confirming that severe asthma can present in different ways from traditional asthma. Despite the fact that the meaning of the term severe asthma remains vague, severe asthma includes existing disease entities: steroid-insensitive asthma, steroid-resistant asthma, and refractory asthma. These subsets of asthmatics seem to constitute up to 5–10% of all asthmatics^42^. Since current treatment guidelines for asthma are based on the very typical phenotypic group of Th2-predominant asthma^43^, the unmet needs of current therapies comprise better treatment of patients with severe asthma. In particular, severe and fatal asthma has seemed to be mediated by neutrophils^44^,^45^. Considering this concept, we used an animal model for severe asthma, specifically steroid-resistant neutrophilic asthma^46^, in this study. Therefore, we focused on the neutrophil recruitment in BALF rather than eosinophils or mast cells, which are more associated with Th2 inflammatory responses of typical asthma. It is well known that inflammation of the asthmatic airway is usually accompanied by increased vascular permeability and plasma exudation^47^. Various inflammatory cytokines, including Th2 cytokines and chemokines such as keratinocyte-derived chemokine (KC), can promote microvascular leakage^48^. The plasma protein leakage induces a thickened, engorged, and edematous airway wall, resulting in the airway lumen narrowing and profound alterations in the extracellular matrix, which are linked to airway hyper-responsiveness and airway resistance in asthma. Supporting these observations, our previous studies have demonstrated that a representative vascular permeability factor, vascular endothelial growth factor (VEGF), plays a critical role in the development of airway inflammation and airway hyper-responsiveness in the pathogenesis of asthma^49^,^50^,^51^,^52^. Taken together, it appears that the pharmacological effect of LC28-0126 on airway hyper-responsiveness caused by the suppression of inflammatory responses and plasma exudation, not by a direct smooth muscle relaxation in this experimental model.

Nevertheless, the Connectivity Map does not provide a comprehensive catalog to denote all possible cell types, pathological conditions, and perturbagens. These problems limit its application to find new indications and/or therapeutics based on drug based computational repositioning. In spite of this limitation, this study demonstrated that even an incomplete collection of the Connectivity Map is useful for finding new clinical uses of investigational drugs. Growing the collection would widen the application of the Connectivity Map for the repositioning of existing drugs and/or identification of possible indications of investigational drugs.

Conclusively, we demonstrated a potential general utility of the computational drug repositioning using the clinical profile of an investigational drug at Phase 1 clinical trial. Through this innovative approach, LC28-0126 was inferred as a potential therapeutics for bronchial asthma. LC28-0126 effectively ameliorates pathophysiology of neutrophilic bronchial asthma in OVA_LPS_-OVA mice. Because the preclinical safety and clinical pharmacokinetics of LC28-0126 are already established, this will facilitate clinical investigation of LC28-0126 for treating asthma in human subjects.

## Methods

### Guideline compliance

All of the methods that are described in this study were carried out in accordance with the approved guidelines for the use of experimental animals and human subjects.

### Human specimen

The methods described below were carried out in accordance with the approved guidelines. This study has been approved by the Institutional Review Board at Seoul National University Hospital, Korea and the Ministry of Food and Drug Safety in Korea (Protocol No. LG-CYCL001). Human specimens were obtained from a study to investigate the safety and pharmacokinetic characteristics of LC28-0126 in healthy male subjects (NCT01737424). Informed consent was obtained from all patients. Male subjects with a body mass index between 18 to 27 kg/m^2^, a total body weight between 55 to 90 kg, and regular alcohol consumption of less than 21 units per week were included. Among the male subjects eligible for this study, 8 subjects were randomly assigned per dose levels of LC28-0126 at 1, 3, 10, or 25 mg per person (32 subjects in total). Blood samples were collected before and 6 hrs after intravenous infusion of LC28-0126. One mL of total blood samples was harvested in a heparin-coated, 1.5 mL centrifuge tube and sera were isolated by centrifugation. Eight mLs of whole blood were harvested in a CPT tube with sodium citrate (BD Biosciences, USA), and peripheral blood mononuclear cells (PBMCs) were isolated by centrifugation according to the manufacturer’s instruction. Isolated sera and PBMCs were kept at −20 °C until use (Supplementary Table S5).

### Cytometrix bead array (CBA)

Five of 8 sera from each dose level of LC28-0126 were randomly selected for cytokine profiling. Inflammatory cytokines (IL-8, IL-1β, IL-6, IL-10, TNF, and IL-12p70) and Th1, Th2, and Th17 cytokines (IL-2, IL-4, IL-17A, and IFN-γ) were measured by using CBA Th1/Th2/Th17 Kit (BD Biosciences, USA) according to the manufacturer’s instruction. Data were analyzed in FCAP Array software (BD Biosciences).

For statistical analysis of cytokine profiles, we used GraphPad Prism 4 statistical software (version 4.01, GraphPad Software Inc., USA). Data were expressed as mean ± SD. Statistical comparisons were performed using a one-sided t-test. A value of *p* < 0.05 was considered statistically significant.

### GeneChip array and differentially expressed genes (DEGs) analysis

Total RNA from PBMCs was extracted by using RNeasy mini kit (Qiagen, USA). RNA purity and concentration were measured by NanoDrop (Thermo scientific, USA), and RNA integrity by 2100 Bioanalyzer (Agilent technology, USA). RNA samples per each dose level of LC28-0126 were pooled before *in vitro* transcription (IVT). Biotin-labeled antisense RNA was prepared by using GeneChip HT 3’IVT Expression Kit (Affymetrix, USA) according to the manufacturer’s instruction. Global gene expression was analyzed by using GeneChip Human Genome 133 Plus 2.0 Array (Affymetrix). After GeneChip scanning, quality of image border, signal intensity (noise ≤5, background 20% to 100%), signal consistency, hybridization process, and labeling process were inspected according to the manufacturer’s instruction. Signal intensities were then subjected to preliminary analyses by box plotting, correlation matrix plotting, and correlation scatter plotting (Supplementary Fig. S1).

Signal intensities were normalized by using Robust Multi-array Average (RMA) normalization. Normalized signal intensities were applied to ComparativeMarkerSelection in GenePattern^53^ to identify differentially expressed genes (DEGs) by LC28-0126. The test was conducted using a 2-sided t-test with 10,000 permutations. DEGs were identified as follows: Present call in more than 75% of samples, coefficient of variance ≤0.41 at 0 hr representing intrinsic threshold of expressional variation of human genes, fold change (6 hrs/0 hr) ≥2, expression pattern = same direction of regulation of at least 3 dose levels of LC28-0126, including 1 dose level satisfying all above conditions. Signaling pathway analysis and functional annotation of DEGs were performed using MetaCore (Thomson Reuters, USA), an integrated knowledge database and software suite for functional analysis of genes, proteins and metabolites.

### Statistically significant connection’s map (sscMap) analysis

Additional therapeutic use of LC28-0126 was identified in comparison with the reference drug profiles in the Connectivity Map^16^. The Connectivity Map consists of 6,100 gene expression profiles of 1,309 US FDA approved and/or experimental drugs. Gene expression profiles were obtained from 5 human cancer cell lines including MCF7, ssMCF7, PC3, HL60, and SKMEL5 at dose levels from 1 nM to 100 μM. Statistically significant connections between LC28-0126 and reference drugs were identified through sscMap^17^, an enhanced sensitivity and principled statistical procedure in mapping the meaningful connections. DEGs byLC28-0126 were used as a query gene signature and applied to sscMap (http://purl.oclc.org/NET/sscMap). Connectivity score was calculated based on signature rank and gene rank to the reference profiles, and significant connection was identified at *p*-value cutoff = 0.05, standardized score cutoff = |3|, and false connection cutoff = query length × 0.2.

### Preclinical validation of anti-asthmatic effect of LC28-0126

The animal study has been approved by the Institutional Animal Care and Use Committee of Chonbuk National University. Anti-asthmatic effect of LC28-0126 was evaluated in an acute neutrophilic asthma mouse model^46^. Briefly, murine-specific pathogens-free female C57BL/6 mice at 6 to 8 weeks old were obtained from the Orient Bio Inc. (Korea). They were housed throughout the experiments in a laminar flow cabinet, and were maintained on standard laboratory chow *ad libitum*. Mice were sensitized intranasally with 10 μg of ovalbumin (OVA; Sigma-Aldrich, USA) with 1 μg of lipopolysaccharide (LPS; Sigma-Aldrich) on days 1 to 3 and 14, followed by challenge with 3% (w/v) OVA inhalation for 30 min using an ultrasonic nebulizer (Omron, Japan) on days 21 to 23 to induce OVA_LPS_-OVA mice. LC28-0126 at 3 or 30 mg/kg bodyweight was intravenously administered twice, 1 hr before the first challenge and 6 hrs after the last challenge. Animals were humanely sacrificed on day 25.

From BALF, total cell count was assessed with NucleoCounter (Chemometec, Denmark). Differential cell count was performed after Diff-Quik staining (Dade Diagnostics of Puerto Rico Inc., Puerto Rico), and approximately 400 cells were counted for the cell differentials.

Expression levels of inflammatory cytokines and chemokines in the lung tissues were assessed with Western blotting. Blots were incubated with and anti-IL-4 antibody (Serotec Ltd., UK), anti-IL-5 antibody (Santa Cruz Biotechnology, USA), anti-IL-13 antibody (R&D systems, USA), anti-IL-17 antibody (R&D systems), anti-KC antibody (BioVision, USA), and anti-actin antibody (Sigma-Aldrich). Relative intensity of each band was quantified using MultiGauge software (Fujifilm, Japan).

Airway responsiveness was assessed at 48 hrs after the last challenge. Unrestrained and conscious mice were placed in a barometric plethysmographic chamber (Allmedicus, Korea) and exposed to aerosolized methacholine (2.5 to 50 mg/mL). Enhanced pause (Penh) was calculated as (expiratory time/relaxation time –1) × (peak expiratory flow/peak inspiratory flow) according to the manufacturer’s instruction.

Formalin-fixed, paraffin embedded lung and trachea were cut at 4 μm sections, placed on slide glass, and subjected to hematoxylin 2 and eosin-Y staining (Richard-Allan Scientific, USA). Histopathological examination was performed under a light microscope (Axio Imager M1, Karl Zeiss, Germany) under identical conditions, including magnification (×20), gain, camera position, and background illumination^54^.

For statistical analysis of preclinical experimental data, we used SPSS statistical software (version 18.0, SPSS, USA). Data were expressed as mean ± SEM. Statistical comparisons were performed using one-way ANOVA followed by the Scheffe’s test. A value of *p* < 0.05 was considered statistically significant.

### 28-day intravenous repeat dose toxicity study of LC28-0126

The animal study has been approved by the Institutional Animal Care and Use Committee of the LG Life Sciences R&D Park. Male and female ICR mice at 5 weeks old were purchased from Orient Bio Inc., housed throughout the experiments in a laminar flow cabinet, and were maintained on standard laboratory chow *ad libitum*. After 1-week of acclimation, 10 animals of each sex were designated to LC28-0126 at 0, 40, or 80 mg/kg/day, respectively. LC28-0126 was injected intravenously (bolus) once daily to 6-week-old ICR mice for 28 days. Toxicity parameters including body weight, hematology, organ weights, and histopathology were evaluated after completion of the treatment.

## Additional Information

**How to cite this article**: Shin, E. *et al.* Drug Signature-based Finding of Additional Clinical Use of LC28-0126 for Neutrophilic Bronchial Asthma. *Sci. Rep.* **5**, 17784; doi: 10.1038/srep17784 (2015).

## Supplementary Material

Supplementary Information

## Figures and Tables

**Table 1:**
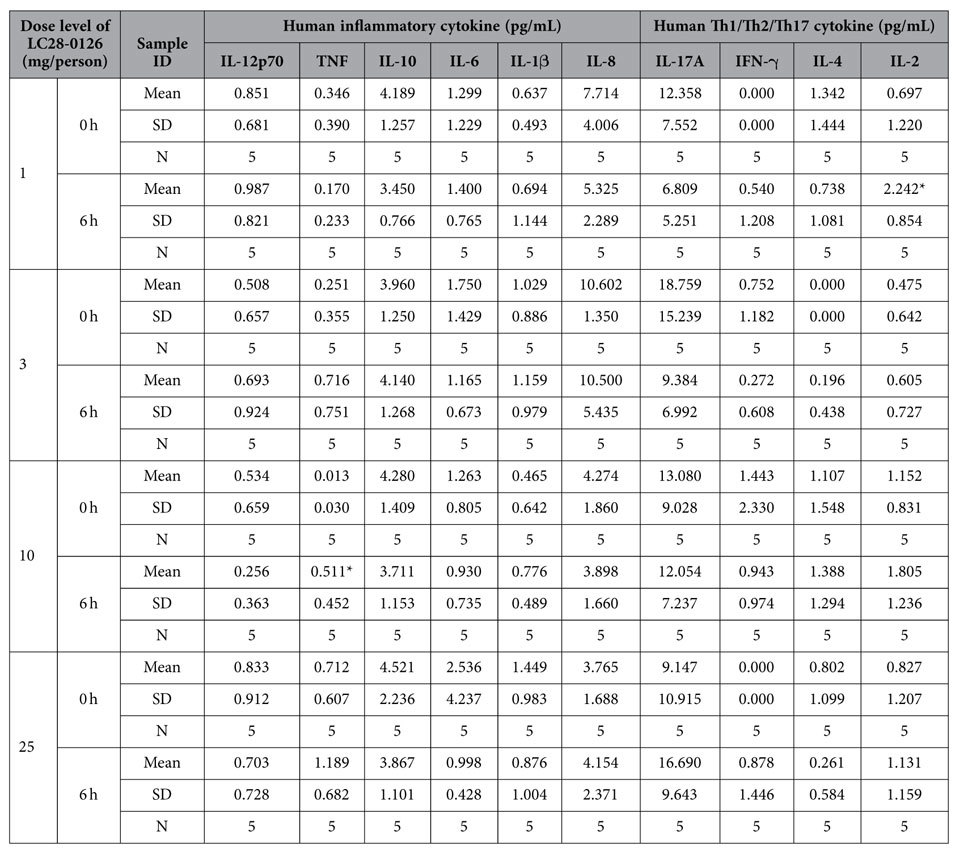
Cytokine profile of LC28-0126 in healthy subjects.

^*^p < 0.05, one-sided t-test.

**Table 2:** Representative differentially expressed genes by LC28-0126 in healthy subjects.

Gene symbol	Gene name	Protein function	CV^1^ at 0 hr	Signaling pathway	Fold change(6 hr/0 hr)	*p* value^2^
*PLA2G4A*	phospholipase A2, group IVA (cytosolic, calcium-dependent)	Generic phospholipase	0.241	Arachidonic acid metabolism	1.55	0.0365
*ADRB1*	adrenergic, beta-1-, receptor	Generic receptor	0.216	β-adrenergic regulation	1.96	0.0133
*CD36*	CD36 molecule (thrombospondin receptor)	Generic receptor	0.356	Oxidative stress	1.71	0.0844
*PDK4*	pyruvate dehydrogenase kinase, isozyme 4	Protein kinase	0.171	PDH-dependent glucose oxidation	−3.31	0.0002

^1^Coefficient of variation

^2^*p* value between 0 hr and 6 hr, two-sided t-test.

**Table 3:** Top ranked statistically significant connections of LC28-0126 with reference drug profiles.

ATC^1^	ATC classification	CMAP instance	Concentration	Cell line	Set size	Query length	Score	*P* value	Standardized score
R03DA01	DRUGS FOR OBSTRUCTIVE AIRWAY DISEASES	Dyphilline	15.8 uM	MCF7	2	24	0.3728	0.00010	4.1185
N07CA01	OTHER NERVOUS SYSTEM DRUGS	Betahistine	17.2 uM	PC3	1	24	0.4484	0.00004	3.8125
G04AB03	UROLOGICALS	Pipemidic Acid	13.2 uM	MCF7	1	24	0.4005	0.00034	3.4050
N/A^2^		5186223	12 uM	MCF7	1	24	0.3914	0.00048	3.3274
C01BC04	CARDIAC THERAPY	Flecainide	8.4 uM	PC3	2	24	0.1995	0.00100	3.2787
C05AA11	VASOPROTECTIVES	Fluocinonide	8 uM	MCF7	3	24	−0.3693	0.00010	−3.9616
R05DB21	COUGH AND COLD PREPARATIONS	Cloperastine	11 uM	MCF7	3	24	−0.2523	0.00020	−3.9635
N/A		Sirolimus	0.1 uM	MCF7	2	24	−0.4078	0.00010	−4.0492
C01BC03	CARDIAC THERAPY	Propafenone	10.6 uM	MCF7	2	24	−0.3833	0.00010	−4.0797
A07EA07	ANTIDIARRHEALS, INTESTINAL ANTIINFLAMMATORY/ANTIINFECTIVE AGENTS	Beclometasone	7.6 uM	MCF7	1	24	−0.4967	0.00001	−4.2229

^1^ATC: Anatomical Therapeutic Chemical Classification System.

^2^N/A: Not Available.

## References

[b1] Booth B. & Zemmel R. Prospects for productivity. Nature reviews. Drug discovery 3, 451–456, 10.1038/nrd1384 (2004). 15136792

[b2] Schulze U., Baedeker M., Chen Y. T. & Greber D. R&D productivity: on the comeback trail. Nature reviews. Drug discovery 13, 331–332, 10.1038/nrd4320 (2014). 24751818

[b3] Chong C. R. & Sullivan D. J. Jr. New uses for old drugs. Nature 448, 645–646, 10.1038/448645a (2007). 17687303

[b4] Liu Z. *et al.* In silico drug repositioning: what we need to know. Drug discovery today 18, 110–115, 10.1016/j.drudis.2012.08.005 (2013). 22935104

[b5] Dudley J. T., Deshpande T. & Butte A. J. Exploiting drug-disease relationships for computational drug repositioning. Briefings in bioinformatics 12, 303–311, 10.1093/bib/bbr013 (2011). 21690101 PMC3137933

[b6] Hu G. & Agarwal P. Human disease-drug network based on genomic expression profiles. PloS one 4, e6536, 10.1371/journal.pone.0006536 (2009). 19657382 PMC2715883

[b7] Campillos M., Kuhn M., Gavin A. C., Jensen L. J. & Bork P. Drug target identification using side-effect similarity. Science 321, 263–266, 10.1126/science.1158140 (2008). 18621671

[b8] Dudley J. T. *et al.* Computational repositioning of the anticonvulsant topiramate for inflammatory bowel disease. Science translational medicine 3, 96ra76, 10.1126/scitranslmed.3002648 (2011). PMC3479650 21849664

[b9] Eckert H. & Bajorath J. Molecular similarity analysis in virtual screening: foundations, limitations and novel approaches. Drug discovery today 12, 225–233, 10.1016/j.drudis.2007.01.011 (2007). 17331887

[b10] Zahler S. *et al.* Inverse in silico screening for identification of kinase inhibitor targets. Chemistry & biology 14, 1207–1214, 10.1016/j.chembiol.2007.10.010 (2007). 18022559

[b11] Kim H. J. *et al.* NecroX as a novel class of mitochondrial reactive oxygen species and ONOO(-) scavenger. Archives of pharmacal research 33, 1813–1823, 10.1007/s12272-010-1114-4 (2010). 21116785

[b12] Chung H. K. *et al.* The indole derivative NecroX-7 improves nonalcoholic steatohepatitis in ob/ob mice through suppression of mitochondrial ROS/RNS and inflammation. Liver international: official journal of the International Association for the Study of the Liver 35, 1341–1353, 10.1111/liv.12741 (2015). 25443620

[b13] Park J. *et al.* NecroX-7 prevents oxidative stress-induced cardiomyopathy by inhibition of NADPH oxidase activity in rats. Toxicology and applied pharmacology 263, 1–6, 10.1016/j.taap.2012.05.014 (2012). 22659508

[b14] Kim H. J. *et al.* A novel small molecule, NecroX-7, inhibits osteoclast differentiation by suppressing NF-kappaB activity and c-Fos expression. Life sciences 91, 928–934, 10.1016/j.lfs.2012.09.009 (2012). 23000100

[b15] Im K. I. *et al.* The Free Radical Scavenger NecroX-7 Attenuates Acute Graft-versus-Host Disease via Reciprocal Regulation of Th1/Regulatory T Cells and Inhibition of HMGB1 Release. Journal of immunology 194, 5223–5232, 10.4049/jimmunol.1402609 (2015). PMC4432727 25911749

[b16] Lamb J. *et al.* The Connectivity Map: using gene-expression signatures to connect small molecules, genes, and disease. Science 313, 1929–1935, 10.1126/science.1132939 (2006). 17008526

[b17] Zhang S. D. & Gant T. W. sscMap: an extensible Java application for connecting small-molecule drugs using gene-expression signatures. BMC bioinformatics 10, 236, 10.1186/1471-2105-10-236 (2009). 19646231 PMC2732627

[b18] Birnbaum Y. *et al.* Prostaglandins mediate the cardioprotective effects of atorvastatin against ischemia-reperfusion injury. Cardiovascular research 65, 345–355, 10.1016/j.cardiores.2004.10.018 (2005). 15639473

[b19] Birnbaum Y., Long B., Qian J., Perez-Polo J. R. & Ye Y. Pioglitazone limits myocardial infarct size, activates Akt, and upregulates cPLA2 and COX-2 in a PPAR-gamma-independent manner. Basic research in cardiology 106, 431–446, 10.1007/s00395-011-0162-3 (2011). 21360043

[b20] Kerkela R. *et al.* Cytosolic phospholipase A(2)alpha protects against ischemia/reperfusion injury in the heart. Clinical and translational science 4, 236–242, 10.1111/j.1752-8062.2011.00294.x (2011). 21884509 PMC3170125

[b21] Lameris T. W. *et al.* Time course and mechanism of myocardial catecholamine release during transient ischemia *in vivo*. Circulation 101, 2645–2650 (2000). 10840018 10.1161/01.cir.101.22.2645

[b22] Murry C. E., Jennings R. B. & Reimer K. A. Preconditioning with ischemia: a delay of lethal cell injury in ischemic myocardium. Circulation 74, 1124–1136 (1986). 3769170 10.1161/01.cir.74.5.1124

[b23] Frances C. *et al.* Role of beta 1- and beta 2-adrenoceptor subtypes in preconditioning against myocardial dysfunction after ischemia and reperfusion. Journal of cardiovascular pharmacology 41, 396–405 (2003). 12605018 10.1097/00005344-200303000-00008

[b24] Spear J. F. *et al.* beta1-Adrenoreceptor activation contributes to ischemia-reperfusion damage as well as playing a role in ischemic preconditioning. American journal of physiology. Heart and circulatory physiology 292, H2459–2466, 10.1152/ajpheart.00459.2006 (2007). 17237252

[b25] Neckar J. *et al.* CD36 overexpression predisposes to arrhythmias but reduces infarct size in spontaneously hypertensive rats: gene expression profile analysis. Physiological genomics 44, 173–182, 10.1152/physiolgenomics.00083.2011 (2012). 22128087 PMC3289117

[b26] Irie H. *et al.* Myocardial recovery from ischemia is impaired in CD36-null mice and restored by myocyte CD36 expression or medium-chain fatty acids. Proceedings of the National Academy of Sciences of the United States of America 100, 6819–6824, 10.1073/pnas.1132094100 (2003). 12746501 PMC164530

[b27] Tanaka T. *et al.* Defect in human myocardial long-chain fatty acid uptake is caused by FAT/CD36 mutations. Journal of lipid research 42, 751–759 (2001). 11352982

[b28] Sugden M. C. & Holness M. J. Recent advances in mechanisms regulating glucose oxidation at the level of the pyruvate dehydrogenase complex by PDKs. American journal of physiology. Endocrinology and metabolism 284, E855–862, 10.1152/ajpendo.00526.2002 (2003). 12676647

[b29] Teague W. M., Pettit F. H., Wu T. L., Silberman S. R. & Reed L. J. Purification and properties of pyruvate dehydrogenase phosphatase from bovine heart and kidney. Biochemistry 21, 5585–5592 (1982). 6293549 10.1021/bi00265a031

[b30] Yeaman S. J. *et al.* Sites of phosphorylation on pyruvate dehydrogenase from bovine kidney and heart. Biochemistry 17, 2364–2370 (1978). 678513 10.1021/bi00605a017

[b31] Pilegaard H. & Neufer P. D. Transcriptional regulation of pyruvate dehydrogenase kinase 4 in skeletal muscle during and after exercise. The Proceedings of the Nutrition Society 63, 221–226 (2004). 15294034 10.1079/pns2004345

[b32] Marketou M. *et al.* Cardioprotective effects of a selective B(2) receptor agonist of bradykinin post-acute myocardial infarct. American journal of hypertension 23, 562–568, 10.1038/ajh.2010.20 (2010). 20186129

[b33] Peters-Golden M., Canetti C., Mancuso P. & Coffey M. J. Leukotrienes: underappreciated mediators of innate immune responses. Journal of immunology 174, 589–594 (2005). 10.4049/jimmunol.174.2.589 15634873

[b34] Barnes P. J. Glucocorticosteroids: current and future directions. British journal of pharmacology 163, 29–43, 10.1111/j.1476-5381.2010.01199.x (2011). 21198556 PMC3085866

[b35] McCord J. M. Superoxide dismutase, lipid peroxidation, and bell-shaped dose response curves. Dose-response: a publication of International Hormesis Society 6, 223–238, 10.2203/dose-response.08-012.McCord (2008). 18846257 PMC2564759

[b36] Noble M., Mayer-Proschel M. & Proschel C. Redox regulation of precursor cell function: insights and paradoxes. Antioxidants & redox signaling 7, 1456–1467, 10.1089/ars.2005.7.1456 (2005). 16356108

[b37] Kawagishi H. & Finkel T. Unraveling the truth about antioxidants: ROS and disease: finding the right balance. Nature medicine 20, 711–713, 10.1038/nm.3625 (2014). 24999942

[b38] Iorio F., Rittman T., Ge H., Menden M. & Saez-Rodriguez J. Transcriptional data: a new gateway to drug repositioning? Drug discovery today 18, 350–357, 10.1016/j.drudis.2012.07.014 (2013). 22897878 PMC3625109

[b39] Turpaev K. T. Reactive oxygen species and regulation of gene expression. Biochemistry. Biokhimiia 67, 281–292 (2002). 11970728 10.1023/a:1014819832003

[b40] Galli S. J., Tsai M. & Piliponsky A. M. The development of allergic inflammation. Nature 454, 445–454, 10.1038/nature07204 (2008). 18650915 PMC3573758

[b41] Holgate S. T. A look at the pathogenesis of asthma: the need for a change in direction. Discovery medicine 9, 439–447 (2010). 20515612

[b42] Barnes P. J. Severe asthma: advances in current management and future therapy. The Journal of allergy and clinical immunology 129, 48–59, 10.1016/j.jaci.2011.11.006 (2012). 22196524

[b43] Shirtcliffe P., Weatherall M., Travers J. & Beasley R. The multiple dimensions of airways disease: targeting treatment to clinical phenotypes. Current opinion in pulmonary medicine 17, 72–78, 10.1097/MCP.0b013e328341f181 (2011). 21150622

[b44] Lamblin C. *et al.* Bronchial neutrophilia in patients with noninfectious status asthmaticus. American journal of respiratory and critical care medicine 157, 394–402, 10.1164/ajrccm.157.2.97-02099 (1998). 9476849

[b45] Tsokos M. & Paulsen F. Expression of pulmonary lactoferrin in sudden-onset and slow-onset asthma with fatal outcome. Virchows Archiv: an international journal of pathology 441, 494–499, 10.1007/s00428-002-0666-1 (2002). 12447681

[b46] Kim S. R. *et al.* Endoplasmic reticulum stress influences bronchial asthma pathogenesis by modulating nuclear factor kappaB activation. The Journal of allergy and clinical immunology 132, 1397–1408, 10.1016/j.jaci.2013.08.041 (2013). 24161747

[b47] Bousquet J., Jeffery P. K., Busse W. W., Johnson M. & Vignola A. M. Asthma. From bronchoconstriction to airways inflammation and remodeling. American journal of respiratory and critical care medicine 161, 1720–1745, 10.1164/ajrccm.161.5.9903102 (2000). 10806180

[b48] Lee C. G. *et al.* Vascular endothelial growth factor (VEGF) induces remodeling and enhances TH2-mediated sensitization and inflammation in the lung. Nature medicine 10, 1095–1103, 10.1038/nm1105 (2004). PMC3434232 15378055

[b49] Lee K. S. *et al.* Vascular endothelial growth factor modulates matrix metalloproteinase-9 expression in asthma. American journal of respiratory and critical care medicine 174, 161–170, 10.1164/rccm.200510-1558OC (2006). 16645174

[b50] Lee K. S. *et al.* Mast cells can mediate vascular permeability through regulation of the PI3K-HIF-1alpha-VEGF axis. American journal of respiratory and critical care medicine 178, 787–797, 10.1164/rccm.200801-008OC (2008). 18669818

[b51] Kim S. R. *et al.* Involvement of sirtuin 1 in airway inflammation and hyperresponsiveness of allergic airway disease. The Journal of allergy and clinical immunology 125, 449-460 e414, 10.1016/j.jaci.2009.08.009 (2010). 19864008

[b52] Kim S. R., Lee K. S., Lee K. B. & Lee Y. C. Recombinant IGFBP-3 inhibits allergic lung inflammation, VEGF production, and vascular leak in a mouse model of asthma. Allergy 67, 869–877, 10.1111/j.1398-9995.2012.02837.x (2012). 22563687

[b53] Reich M. *et al.* GenePattern 2.0. Nature genetics 38, 500–501, 10.1038/ng0506-500 (2006). 16642009

[b54] Cho J. Y. *et al.* Inhibition of airway remodeling in IL-5-deficient mice. The Journal of clinical investigation 113, 551–560, 10.1172/JCI19133 (2004). 14966564 PMC338264

