# Supplementary material for: Drug Signature-based Finding of Additional Clinical Use of LC28-0126 for Neutrophilic Bronchial Asthma
Source: Sci Rep. 2015 Dec 2;5:17784. doi: 10.1038/srep17784 (PMC4667219; doi:10.1038/srep17784)

Drug Signature-based Finding of Additional Clinical Use of LC28-0126 For Neutrophilic Bronchial Asthma

Authors: Eunji Shin[1], Yong Chul Lee[2], So Ri Kim[2], Soon Ha Kim[1], Joonghoon Park[1]

Affiliations: [1]LG Life Sciences R&D Park, Daejeon, 305-380, Republic of Korea.
[2]Department of Internal Medicine, Research Center for Pulmonary Disorder, Chonbuk National University Medical School, Research Institute of Clinical Medicine of Chonbuk National University-Biomedical Research Institute of Chonbuk National University Hospital, Jeonju, 561-180, Republic of Korea

Correspondence: Joonghoon Park, LG Life Sciences R&D Park, Daejeon, 305-380, Republic of Korea. E-mail: joonghoon@lgls.com

Supplementary Information

Supplementary Table S1. Individual cytokine profile before and after LC28-0126 infusion
Supplementary Table S2. Differentially expressed genes 6 hrs after LC28-0126 infusion
Supplementary Table S3. Full list of statistically significant connections of LC28-0126
Supplementary Table S4. Summary of 28-day repeat dose toxicity study of LC28-0126 in ICR mice
Supplementary Table S5. Summary of sample information, RNA quality control, and uses thereof

Supplementary Fig. S1. Preliminary microarray data analysis. (A) Inter-quartile range of signal intensities (B) Spearman rank correlation coefficient matrix among GeneChip (C) Spearman correlation coefficient scatter plot among GeneChip

Supplementary Table S1. Individual cytokine profile before and after LC28-0126 infusion

Dose level of LC28-0126 (mg/person)	Sample ID	Human inflammatory cytokine (pg/mL)						Human Th1/Th2/Th17 cytokine kit (pg/mL)			
		IL-12p70	TNF	IL-10	IL-6	IL-1β	IL-8	IL-17A	IFN-γ	IL-4	IL-2
1	R7201-0h	0.000	0.000	2.166	0.555	0.000	7.615	19.432	0.000	0.000	2.816
	R7202-0h	0.771	0.345	5.643	1.566	0.328	13.731	17.575	0.000	3.365	0.000
	R7204-0h	1.897	0.000	4.341	1.148	1.062	6.081	9.351	0.000	2.137	0.000
	R7206-0h	0.683	0.441	4.455	3.223	0.617	2.733	14.739	0.000	1.207	0.000
	R7208-0h	0.905	0.945	4.341	0.000	1.176	8.412	0.690	0.000	0.000	0.669
	R7201-6h	0.000	0.000	2.602	0.471	0.000	5.417	0.000	0.000	0.000	0.930
	R7202-6h	1.750	0.408	3.534	2.525	2.634	8.795	5.748	0.000	1.305	2.440
	R7204-6h	1.799	0.000	4.680	1.614	0.000	2.837	12.324	0.000	0.000	3.290
	R7206-6h	0.301	0.441	3.217	1.402	0.838	3.754	4.140	2.700	0.000	2.440
	R7208-6h	1.087	0.000	3.217	0.990	0.000	5.822	11.834	0.000	2.386	2.110
3	R1201-0h	0.000	0.000	2.166	0.000	0.187	12.300	39.726	0.000	0.000	1.421
	R1202-0h	1.604	0.408	4.455	1.263	1.637	9.834	25.749	2.700	0.000	0.866
	R1203-0h	0.511	0.000	5.606	3.918	0.436	11.826	0.000	0.000	0.000	0.000
	R1205-0h	0.426	0.038	3.690	2.074	0.617	9.487	18.971	1.058	0.000	0.000
	R1208-0h	0.000	0.807	3.884	1.496	2.270	9.564	9.351	0.000	0.000	0.087
	R1201-6h	1.799	1.786	2.029	0.000	0.000	9.332	0.000	0.000	0.000	1.302
	R1202-6h	0.000	1.121	4.266	1.566	1.521	4.616	5.748	0.000	0.979	0.000
	R1203-6h	0.000	0.067	5.459	1.638	1.405	19.379	13.779	1.359	0.000	0.000
	R1205-6h	0.060	0.604	4.455	1.194	2.472	10.494	9.351	0.000	0.000	0.243
	R1208-6h	1.604	0.000	4.492	1.425	0.400	8.680	18.042	0.000	0.000	1.481
10	R2201-0h	0.000	0.000	3.573	0.000	0.000	1.871	20.809	0.000	0.000	0.318
	R2202-0h	0.426	0.000	6.591	1.976	0.000	4.544	23.075	5.353	3.212	2.110
	R2203-0h	0.640	0.067	3.056	1.638	0.328	3.047	5.748	0.000	0.000	0.930
	R2205-0h	0.000	0.000	3.573	0.946	1.559	5.307	13.296	0.000	0.000	1.942
	R2206-0h	1.604	0.000	4.605	1.758	0.436	6.601	2.470	1.860	2.325	0.463
	R2201-6h	0.000	0.000	1.936	0.000	1.290	1.430	16.165	1.359	0.000	0.000
	R2202-6h	0.511	0.506	5.164	1.976	0.653	4.870	15.216	1.058	2.006	1.119
	R2203-6h	0.000	1.229	3.884	1.125	0.949	2.942	0.000	0.000	0.000	2.330
	R2205-6h	0.000	0.313	3.690	1.013	0.000	5.307	10.848	2.296	2.201	3.081
	R2206-6h	0.771	0.506	3.884	0.534	0.987	4.943	18.042	0.000	2.732	2.494
25	R3201-0h	0.000	0.000	1.793	0.619	0.000	1.028	0.000	0.000	0.000	0.000
	R3203-0h	2.196	0.313	7.782	10.031	1.637	4.544	16.637	0.000	0.000	2.656
	R3205-0h	1.225	1.157	3.297	0.000	1.328	5.307	0.000	0.000	2.006	1.481
	R3207-0h	0.683	1.485	5.164	1.686	2.757	3.329	4.681	0.000	2.006	0.000
	R3208-0h	0.060	0.604	4.567	0.347	1.521	4.616	24.418	0.000	0.000	0.000
	R3201-6h	0.000	0.000	3.097	0.619	0.000	1.161	18.042	0.000	0.000	0.000
	R3203-6h	1.653	1.448	5.127	1.614	1.100	4.652	24.863	3.331	0.000	0.669
	R3205-6h	0.683	1.265	3.922	1.217	0.544	2.698	2.470	0.000	0.000	0.533
	R3207-6h	1.179	1.672	2.474	0.946	2.513	4.833	12.324	1.058	1.305	1.481
	R3208-6h	0.000	1.560	4.717	0.597	0.222	7.426	25.749	0.000	0.000	2.975

Supplementary Table S2. Differentially expressed genes 6 hrs after LC28-0126 infusion

Probe ID	Gene Symbol	Gene Name	Protein Function	Fold change (6 hr/0 hr) at each dose level (mg/person) of LC28-0126			
				1	3	10	25
238692_at	BTBD11	BTB (POZ) domain containing 11, BTBD11	Generic binding protein	-1.05	2.91	1.18	1.04
1554606_at	CEP120	centrosomal protein 120kDa, CEP120	Generic binding protein	-1.17	2.19	1.05	1.27
235918_x_at	CEP97	centrosomal protein 97kDa, CEP97	Generic binding protein	-1.74	1.18	1.10	2.00
231918_s_at	GFM2	G elongation factor, mitochondrial 2, GFM2	Generic binding protein	1.06	2.00	1.10	1.18
229970_at	KBTBD7	---	Generic binding protein	1.10	2.08	1.24	1.97
231875_at	KIF21A	kinesin family member 21A, KIF21A	Generic binding protein	-1.38	1.22	1.01	2.01
233085_s_at	NABP1	oligonucleotide/oligosaccharide-binding fold containing 2A, OBFC2A	Generic binding protein	-1.05	2.12	1.45	1.30
223315_at	NTN4	netrin 4, NTN4	Generic binding protein	1.28	1.47	1.21	2.06
202861_at	PER1	period homolog 1 (Drosophila), PER1	Generic binding protein	-1.43	-1.77	-2.29	-2.10
36829_at	PER1	period homolog 1 (Drosophila), PER1	Generic binding protein	-1.30	-1.48	-2.03	-1.68
204286_s_at	PMAIP1	phorbol-12-myristate-13-acetate-induced protein 1, PMAIP1	Generic binding protein	1.27	2.11	1.49	1.18
213430_at	RUFY3	RUN and FYVE domain containing 3, RUFY3	Generic binding protein	-1.19	1.42	1.37	2.07
232636_at	SLITRK4	SLIT and NTRK-like family, member 4, SLITRK4	Generic binding protein	1.48	2.01	1.67	1.20
219583_s_at	SPATA7	spermatogenesis associated 7, SPATA7	Generic binding protein	1.05	2.00	1.69	1.20
226837_at	SPRED1	sprouty-related, EVH1 domain containing 1, SPRED1	Generic binding protein	1.30	2.34	1.47	1.62
223511_at	SPRTN	chromosome 1 open reading frame 124, C1orf124	Generic binding protein	-1.21	2.18	1.31	1.29
235653_s_at	THAP6	THAP domain containing 6, THAP6	Generic binding protein	1.44	-1.15	1.28	2.03
219330_at	VANGL1	vang-like 1 (van gogh, Drosophila), VANGL1	Generic binding protein	1.16	1.47	-1.00	2.03
235625_at	VPS41	vacuolar protein sorting 41 homolog (S. cerevisiae), VPS41	Generic binding protein	-1.03	2.12	1.44	1.36
206098_at	ZBTB6	zinc finger and BTB domain containing 6, ZBTB6	Generic binding protein	-1.35	2.06	1.61	1.48
1558700_s_at	ZNF260	zinc finger protein 260, ZNF260	Generic binding protein	-1.32	2.43	1.16	1.69
204567_s_at	ABCG1	ATP-binding cassette, sub-family G (WHITE), member 1, ABCG1	Generic channel	1.20	1.72	1.47	2.02
206233_at	B4GALT6	UDP-Gal:betaGlcNAc beta 1,4- galactosyltransferase, polypeptide 6, B4GALT6	Generic enzyme	-1.43	2.09	1.02	1.31
202620_s_at	PLOD2	procollagen-lysine, 2-oxoglutarate 5-dioxygenase 2, PLOD2	Generic enzyme	1.41	1.61	2.02	1.11
232020_at	SMURF2	SMAD specific E3 ubiquitin protein ligase 2, SMURF2	Generic enzyme	-1.25	2.20	1.04	1.59
235334_at	ST6GALNAC3	ST6 (alpha-N-acetyl-neuraminyl-2,3-beta-galactosyl-1,3)-N-acetylgalactosaminide alpha-2,6-sialyltransferase 3, ST6GALNAC3	Generic enzyme	1.28	2.00	1.13	1.03
1556185_a_at	STEAP4	STEAP family member 4, STEAP4	Generic enzyme	1.41	2.56	2.01	1.15
210145_at	PLA2G4A	phospholipase A2, group IVA (cytosolic, calcium-dependent), PLA2G4A	Generic phospholipase	1.34	2.40	1.27	1.19
229309_at	ADRB1	adrenergic, beta-1-, receptor, ADRB1	Generic receptor	1.54	1.66	2.46	2.19
242197_x_at	CD36	CD36 molecule (thrombospondin receptor), CD36	Generic receptor	1.71	1.42	2.08	1.63
235401_s_at	FCRLA	Fc receptor-like A, FCRLA	Generic receptor	-1.25	2.22	1.20	1.30
206995_x_at	SCARF1	scavenger receptor class F, member 1, SCARF1	Generic receptor	1.06	2.12	1.04	1.38
202887_s_at	DDIT4	DNA-damage-inducible transcript 4, DDIT4	Protein	-1.52	-1.65	-2.44	-1.37
219872_at	FAM198B	family with sequence similarity 198, member B, FAM198B	Protein	1.41	2.07	1.13	1.17
213524_s_at	G0S2	G0/G1switch 2, G0S2	Protein	-1.57	-3.26	-1.44	-2.05
225188_at	RAPH1	Ras association (RalGDS/AF-6) and pleckstrin homology domains 1, RAPH1	Protein	1.25	1.70	2.05	2.05
225189_s_at	RAPH1	Ras association (RalGDS/AF-6) and pleckstrin homology domains 1, RAPH1	Protein	1.61	2.29	1.65	1.75
221596_s_at	RBM48	chromosome 7 open reading frame 64, C7orf64	Protein	-1.29	2.07	1.40	1.42
217104_at	ST20	suppressor of tumorigenicity 20, ST20	Protein	1.14	2.02	1.48	1.26
225207_at	PDK4	pyruvate dehydrogenase kinase, isozyme 4, PDK4	Protein kinase	-2.71	-3.72	-2.99	-3.83
204794_at	DUSP2	dual specificity phosphatase 2, DUSP2	Protein phosphatase	-1.38	-2.05	-1.74	-1.19
219787_s_at	ECT2	epithelial cell transforming sequence 2 oncogene, ECT2	Regulators (GDI, GAP, GEF)	-1.06	1.53	1.30	2.28
226122_at	PLEKHG1	pleckstrin homology domain containing, family G (with RhoGef domain) member 1, PLEKHG1	Regulators (GDI, GAP, GEF)	-1.03	2.20	1.04	1.06
204761_at	USP6NL	USP6 N-terminal like, USP6NL	Regulators (GDI, GAP, GEF)	-1.15	2.08	1.01	1.27
203840_at	BLZF1	basic leucine zipper nuclear factor 1, BLZF1	Transcription factor	-1.04	2.05	1.29	1.35
230056_at	BPTF	bromodomain PHD finger transcription factor, BPTF	Transcription factor	-1.19	1.75	1.05	2.09
204621_s_at	NR4A2	nuclear receptor subfamily 4, group A, member 2, NR4A2	Transcription factor	-1.43	-2.04	-1.50	-1.97
204622_x_at	NR4A2	nuclear receptor subfamily 4, group A, member 2, NR4A2	Transcription factor	-1.48	-2.05	-1.32	-1.72
216248_s_at	NR4A2	nuclear receptor subfamily 4, group A, member 2, NR4A2	Transcription factor	-1.81	-2.09	-1.55	-1.76
237388_at	---	---	#N/A	-2.12	-1.15	1.27	-1.07
238913_at	---	---	#N/A	-2.01	-1.14	-1.29	-1.11
242751_at	---	---	#N/A	-2.09	1.02	-1.24	-1.16
243915_at	---	---	#N/A	-2.01	-1.02	1.14	-1.04
227004_at	---	---	#N/A	-1.14	2.02	1.42	1.20
230399_at	---	---	#N/A	-1.28	2.09	1.04	1.23
232958_at	---	---	#N/A	-1.02	2.11	1.40	1.70
235739_at	---	---	#N/A	-1.05	-2.23	-1.05	-1.20
236907_at	---	---	#N/A	-1.76	-2.06	1.08	-1.16
241982_at	---	---	#N/A	-1.17	-2.26	1.18	-1.03
229804_x_at	CBWD	COBW domain containing	#N/A	-1.05	2.08	1.64	1.57
222924_at	LOC100287789	sarcolemma associated protein, SLMAP	#N/A	-1.41	2.11	1.16	1.07
242904_x_at	LOC100653229	---	#N/A	-1.10	-2.04	-1.58	-1.22
1556842_at	LOC286087	---	#N/A	-1.55	-1.19	-2.04	1.28

Supplementary Table S3. Full list of statistically significant connections of LC28-0126

ATC	ATC classification	CMAP instance	Concentration	Cell line	Set size	Query length	Score	P value	Standardized score
R03DA01	DRUGS FOR OBSTRUCTIVE AIRWAY DISEASES	Dyphilline	15.8 uM	MCF7	2	24	0.372823264	0.0001	4.118474448
N07CA01	OTHER NERVOUS SYSTEM DRUGS	Betahistine	17.2 uM	PC3	1	24	0.448413144	4E-05	3.812489077
G04AB03	UROLOGICALS	Pipemidic Acid	13.2 uM	MCF7	1	24	0.400491286	0.00034	3.405048832
#N/A		5186223	12 uM	MCF7	1	24	0.391361531	0.00048	3.327426017
C01BC04	CARDIAC THERAPY	Flecainide	8.4 uM	PC3	2	24	0.19952443	0.001	3.278726625
#N/A		Hemicholinium	7 uM	PC3	1	24	0.382909024	0.00068	3.255561288
#N/A		Yohimbic Acid	11.2 uM	MCF7	1	24	0.373771786	0.00118	3.177874848
#N/A		Estropipate	9.2 uM	MCF7	2	24	0.333840334	0.0008	3.155511554
C01BD01	CARDIAC THERAPY	Amiodarone	5.8 uM	PC3	1	24	-0.370099305	0.00126	-3.146650753
P02CE01	ANTHELMINTICS	Levamisole	16.6 uM	HL60	1	24	-0.371212461	0.00124	-3.15611501
#N/A		Pnu-0230031	10 uM	MCF7	2	24	-0.251049548	0.0008	-3.161011472
C02AA02	ANTIHYPERTENSIVES	Reserpine	6.6 uM	MCF7	1	24	-0.372059957	0.00122	-3.163320571
#N/A		Luteolin	14 uM	MCF7	2	24	-0.346420874	0.001	-3.177249855
R06AX16	ANTIHISTAMINES FOR SYSTEMIC USE	Deptropine	7.6 uM	MCF7	2	24	-0.319828593	0.0006	-3.189901938
D07AC07	CORTICOSTEROIDS, DERMATOLOGICAL PREPARATIONS	Fludroxycortide	9.2 uM	HL60	1	24	-0.375468648	0.00112	-3.192301876
J01FA05	ANTIBACTERIALS FOR SYSTEMIC USE	Oleandomycin	5 uM	PC3	2	24	-0.280896175	0.0008	-3.1933171
N05AF05	PSYCHOLEPTICS	Zuclopenthixol	9.2 uM	PC3	1	24	-0.377571485	0.00104	-3.210180573
#N/A		Clorgiline	13 uM	MCF7	2	24	-0.360741306	0.001	-3.221555282
N05AF01	PSYCHOLEPTICS	Flupentixol	7.8 uM	PC3	1	24	-0.380484401	0.0009	-3.234946705
H02AB12	CORTICOSTEROIDS FOR SYSTEMIC USE	Rimexolone	10.8 uM	MCF7	2	24	-0.326228775	0.0012	-3.246056183
#N/A		Prestwick-1100	9 uM	PC3	2	24	-0.278550128	0.0008	-3.267134262
D07AC17	CORTICOSTEROIDS, DERMATOLOGICAL PREPARATIONS	Fluticasone	8 uM	HL60	1	24	-0.384783617	0.00084	-3.271499415
#N/A		Helveticoside	7.4 uM	MCF7	3	24	-0.303031153	0.0012	-3.277473029
#N/A		0179445-0000	10 uM	PC3	2	24	-0.322285956	0.0006	-3.278298985
D04AB05	ANTIPRURITICS, INCL. ANTIHISTAMINES, ANESTHETICS, ETC.	Quinisocaine	13 uM	MCF7	2	24	-0.326520628	0.0001	-3.280550087
#N/A		Harman	18.2 uM	MCF7	2	24	-0.334290274	0.001	-3.285004179
#N/A		Parbendazole	16.2 uM	PC3	2	24	-0.151229336	0.0012	-3.29131816
#N/A		Blebbistatin	17 uM	MCF7	2	24	-0.313813805	0.001	-3.3174087
N05AD06	PSYCHOLEPTICS	Bromperidol	9.6 uM	MCF7	2	24	-0.357286779	0.0006	-3.32336444
#N/A		Verteporfin	2.8 uM	MCF7	2	24	-0.342555695	0.0006	-3.335359781
#N/A		Suloctidil	11.8 uM	MCF7	2	24	-0.289272912	0.0008	-3.342506094
A03FA02	DRUGS FOR FUNCTIONAL GASTROINTESTINAL DISORDERS	Cisapride	8.6 uM	MCF7	2	24	-0.293348562	0.001	-3.345614747
A03FA01	DRUGS FOR FUNCTIONAL GASTROINTESTINAL DISORDERS	Metoclopramide	11.8 uM	HL60	1	24	-0.393664549	0.00062	-3.34700669
#N/A		Dl-Alpha Tocopherol	9.2 uM	PC3	1	24	-0.393902147	0.00062	-3.349026792
C01AB01	CARDIAC THERAPY	Proscillaridin	7.6 uM	HL60	1	24	-0.396472697	0.00056	-3.370882068
#N/A		Prestwick-559	8.2 uM	MCF7	2	24	-0.367920698	0.0002	-3.381717199
S01BA08	OPHTHALMOLOGICALS	Medrysone	11.6 uM	MCF7	3	24	-0.344465523	0.0008	-3.396629515
C02AA01	ANTIHYPERTENSIVES	Rescinnamine	6.4 uM	MCF7	2	24	-0.311936406	0.0008	-3.45645094
#N/A		Tonzonium Bromide	6.8 uM	PC3	1	24	-0.408438662	0.00034	-3.472618855
N05AC02	PSYCHOLEPTICS	Thioridazine	10 uM	MCF7	8	24	-0.259080879	0.0001	-3.476683635
A02BX06	DRUGS FOR ACID RELATED DISORDERS	Proglumide	12 uM	PC3	2	24	-0.310273219	0.0004	-3.489382892
#N/A		Resveratrol	17.6 uM	MCF7	2	24	-0.345473288	0.0004	-3.492215686
#N/A		0175029-0000	1 uM	PC3	2	24	-0.312709068	0.0001	-3.507815509
#N/A		Thioguanosine	12.6 uM	MCF7	2	24	-0.339074976	0.0001	-3.508472723
P02DA01	ANTHELMINTICS	Niclosamide	12.2 uM	MCF7	1	24	-0.414758398	0.00022	-3.526350386
#N/A		5248896	11 uM	MCF7	2	24	-0.34879592	0.0002	-3.530753994
C03DA01	DIURETICS	Spironolactone	9.6 uM	MCF7	3	24	-0.2244872	0.0002	-3.538286727
J01EA01	ANTIBACTERIALS FOR SYSTEMIC USE	Trimethoprim	13.8 uM	MCF7	2	24	-0.328053791	0.0006	-3.596271582
J01XD02	ANTIBACTERIALS FOR SYSTEMIC USE	Tinidazole	16.2 uM	PC3	2	24	-0.314020534	0.0001	-3.635887299
C08CA02	CALCIUM CHANNEL BLOCKERS	Felodipine	10 uM	MCF7	3	24	-0.244933734	0.0001	-3.646353775
#N/A		Celastrol	2.5 uM	MCF7	1	24	-0.429745789	0.00014	-3.653775871
N04AA04	ANTI-PARKINSON DRUGS	Procyclidine	12.4 uM	MCF7	2	24	-0.343381676	0.0004	-3.660885027
#N/A		Prestwick-674	14.4 uM	MCF7	3	24	-0.386607074	0.0001	-3.666268753
S01FA06	OPHTHALMOLOGICALS	Tropicamide	14 uM	MCF7	3	24	-0.283183291	0.0002	-3.675968527
#N/A		Pararosaniline	10 uM	MCF7	1	24	-0.439466733	0.00006	-3.736425081
#N/A		Epiandrosterone	13.8 uM	HL60	1	24	-0.440772587	0.00006	-3.747527688
#N/A		Resveratrol	10 uM	MCF7	2	24	-0.348536807	0.0006	-3.793269374
B02BA02	ANTIHEMORRHAGICS	Menadione	23.2 uM	PC3	1	24	-0.447982848	0.00006	-3.808830624
C04AX02	PERIPHERAL VASODILATORS	Phenoxybenzamine	11.8 uM	MCF7	3	24	-0.397387543	0.0001	-3.815632773
V03AC01	ALL OTHER THERAPEUTIC PRODUCTS	Deferoxamine	6 uM	MCF7	3	24	-0.341247658	0.0001	-3.948218688
C05AA11	VASOPROTECTIVES	Fluocinonide	8 uM	MCF7	3	24	-0.369289226	0.0001	-3.96157693
R05DB21	COUGH AND COLD PREPARATIONS	Cloperastine	11 uM	MCF7	3	24	-0.252252505	0.0002	-3.963502388
#N/A		Sirolimus	0.1 uM	MCF7	2	24	-0.407839054	0.0001	-4.049208587
C01BC03	CARDIAC THERAPY	Propafenone	10.6 uM	MCF7	2	24	-0.383258873	0.0001	-4.079682355
A07EA07	ANTIDIARRHEALS, INTESTINAL ANTIINFLAMMATORY/ANTIINFECTIVE AGENTS	Beclometasone	7.6 uM	MCF7	1	24	-0.496688593	0.00001	-4.222935615

Supplementary Table S4. Summary of 28-day repeat dose toxicity study of LC28-0126 in ICR mice

		Male			Female		
Dose (mg/kg/day)		0	40	80	0	40	80
No. of animals		10	10	10	10	10	10
Bodyweight at 4-week (g)	Mean	35.5	36.1	35.4	28.3	28.2	28.4
	SD	2.6	2	2.5	2.2	2	1.7
RBC (10^6 cells/uL)	Mean	8.57	8.22	8.48	8.58	8.67	8.48
	SD	0.41	0.43	0.48	0.39	0.24	0.43
WBC (10^3 cells/uL)	Mean	1.77	2.89	3.69	3.05	2.99	2.6
	SD	0.69	1.19	1.46	2.11	1.52	0.86
%Neutrophils	Mean	12	8.8	8.4	8.5	10.9	10.2
	SD	1.2	2.4	2.3	2.1	3	2
%Lymphocytes	Mean	84.4	88.4	89	88.5	85.9	86.4
	SD	1.8	2.9	2.7	3.6	3.8	2.5
%Monocytes	Mean	3.1	2.3	2.3	2.5	2.7	2.8
	SD	0.5	0.5	2.7	1	0.7	1
%Eosinophils	Mean	0.2	0.1	0.1	0.2	0	0.1
	SD	0.2	0.3	0.1	0.3	0.1	0.2
%Basophils	Mean	0.2	0.1	0	0.1	0.1	0.1
	SD	0.1	0.1	0.1	0.1	0.1	0.1
Lung weight (g/100 g bw)	Mean	4.588	4.879	4.955	4.833	5.12	4.95
	SD	0.542	0.425	0.558	0.581	0.611	0.414
Lung inflammation, chronic active, focal	Frequency	0	ND	0	1	ND	1

Supplementary Table S5. Summary of sample information, RNA quality control, and uses thereof

Dose level of LC28-0126 (mg/person)	Sample ID	Age	RNA					Application[2]	
			Conc. (ng/uL)	A260/A280	A260/A230	RIN	Decision[1]	Cytokine	GeneChip
1	R7201-0h	31	234.67	2.10	2.23	9.5	P	D	ND
	R7201-6h		26.48	2.35	1.91	9.1	F	D	ND
	R7202-0h	33	179.33	2.13	2.03	9.2	P	D	ND
	R7202-6h		28.97	2.14	0.77	9.5	F	D	ND
	R7203-0h	25	79.75	2.16	1.74	9.2	P	ND	ND
	R7203-6h		79.29	2.17	2.16	8.9	P	ND	ND
	R7204-0h	30	126.36	2.13	2.17	9.7	P	D	ND
	R7204-6h		87.04	2.20	2.14	9.7	P	D	ND
	R7205-0h	30	103.69	2.12	2.10	9.4	P	ND	ND
	R7205-6h		82.25	2.19	1.35	9.8	P	ND	ND
	R7206-0h	27	146.40	2.12	2.17	9.5	P	D	ND
	R7206-6h		121.21	2.18	2.11	9.6	P	D	ND
	R7207-0h	33	96.05	2.19	1.78	9.7	P	ND	ND
	R7207-6h		83.23	2.16	2.01	9.7	P	ND	ND
	R7208-0h	24	70.84	2.18	2.13	9.7	P	D	ND
	R7208-6h		79.80	2.21	1.39	9.7	P	D	ND
	R7201-7208_0h		111.13	2.05	2.00	9.5	P	ND	D
	R7201-7208_6h		86.53	2.15	1.84	9.7	P	ND	D
3	R1201-0h	25	142.07	2.13	2.19	7.9	F	D	ND
	R1201-6h		284.45	2.08	2.22	9.4	P	D	ND
	R1202-0h	22	320.26	2.08	2.20	9.0	P	D	ND
	R1202-6h		303.68	2.07	2.19	9.4	P	D	ND
	R1203-0h	36	570.67	2.04	2.05	8.9	P	D	ND
	R1203-6h		345.98	2.08	2.21	9.1	P	D	ND
	R1204-0h	22	387.14	2.08	2.16	8.0	P	ND	ND
	R1204-6h		615.90	2.05	2.07	9.2	P	ND	ND
	R1205-0h	28	511.07	2.03	2.08	9.2	P	D	ND
	R1205-6h		363.79	2.08	2.19	9.3	P	D	ND
	R1206-0h	25	327.26	2.09	2.28	9.2	P	ND	ND
	R1206-6h		253.14	2.08	2.25	9.5	P	ND	ND
	R1207-0h	29	478.68	2.02	2.05	9.4	P	ND	ND
	R1207-6h		356.78	2.07	2.01	9.2	P	ND	ND
	R1208-0h	20	217.80	2.09	2.27	9.5	P	D	ND
	R1208-6h		281.43	2.09	2.07	9.3	P	D	ND
	R1201-1208_0h		193.84	2.04	2.28	9.1	P	ND	D
	R1201-1208_6h		159.71	2.04	2.20	9.3	P	ND	D
10	R2201-0h	24	350.36	2.11	2.26	9.0	P	D	ND
	R2201-6h		351.61	2.10	2.20	9.2	P	D	ND
	R2202-0h	26	633.64	2.05	2.16	9.3	P	D	ND
	R2202-6h		476.95	2.01	2.10	9.2	P	D	ND
	R2203-0h	27	218.41	2.08	2.22	9.2	P	D	ND
	R2203-6h		255.80	2.09	2.11	9.4	P	D	ND
	R2204-0h	27	472.16	2.07	2.24	9.5	P	ND	ND
	R2204-6h		338.46	2.09	2.24	9.1	P	ND	ND
	R2205-0h	23	299.95	2.10	2.26	8.8	P	D	ND
	R2205-6h		245.46	2.12	2.24	9.2	P	D	ND
	R2206-0h	26	368.61	2.08	2.24	9.2	P	D	ND
	R2206-6h		212.27	2.13	2.19	9.0	P	D	ND
	R2207-0h	24	396.90	2.09	2.04	8.8	P	ND	ND
	R2207-6h		407.10	2.08	2.25	7.7	F	ND	ND
	R2208-0h	24	315.50	2.10	2.24	8.9	P	ND	ND
	R2208-6h		330.65	2.11	1.39	9.1	P	ND	ND
	R2201-2208_0h		192.46	2.05	2.24	9.1	P	ND	D
	R2201-2208_6h		188.61	2.06	2.05	8.9	P	ND	D
25	R3201-0h	28	441.16	2.09	1.46	9.6	P	D	ND
	R3201-6h		274.19	2.10	2.26	9.5	P	D	ND
	R3202-0h	25	253.82	2.07	2.18	9.3	P	ND	ND
	R3202-6h		185.52	2.13	2.09	8.9	P	ND	ND
	R3203-0h	25	295.69	2.11	2.13	9.1	P	D	ND
	R3203-6h		212.61	2.11	0.90	9.5	P	D	ND
	R3204-0h	28	224.09	2.10	2.17	9.2	P	ND	ND
	R3204-6h		189.15	2.09	1.99	9.0	P	ND	ND
	R3205-0h	20	194.28	2.12	2.23	9.0	P	D	ND
	R3205-6h		259.74	2.08	2.21	9.5	P	D	ND
	R3206-0h	24	170.84	2.12	2.19	9.3	P	ND	ND
	R3206-6h		156.70	2.10	2.23	9.0	P	ND	ND
	R3207-0h	31	241.10	2.10	2.24	9.5	P	D	ND
	R3207-6h		228.71	2.08	2.13	9.3	P	D	ND
	R3208-0h	27	252.14	2.11	2.22	9.5	P	D	ND
	R3208-6h		193.39	2.09	2.20	8.9	P	D	ND
	R3201-3208_0h		173.95	2.03	2.02	9.3	P	ND	D
	R3201-3208_6h		174.29	2.06	1.81	9.4	P	ND	D

[1]P: Passed, F: Failed
[2]D: Determined, ND: Not determined

Supplementary Figure 1 (A)

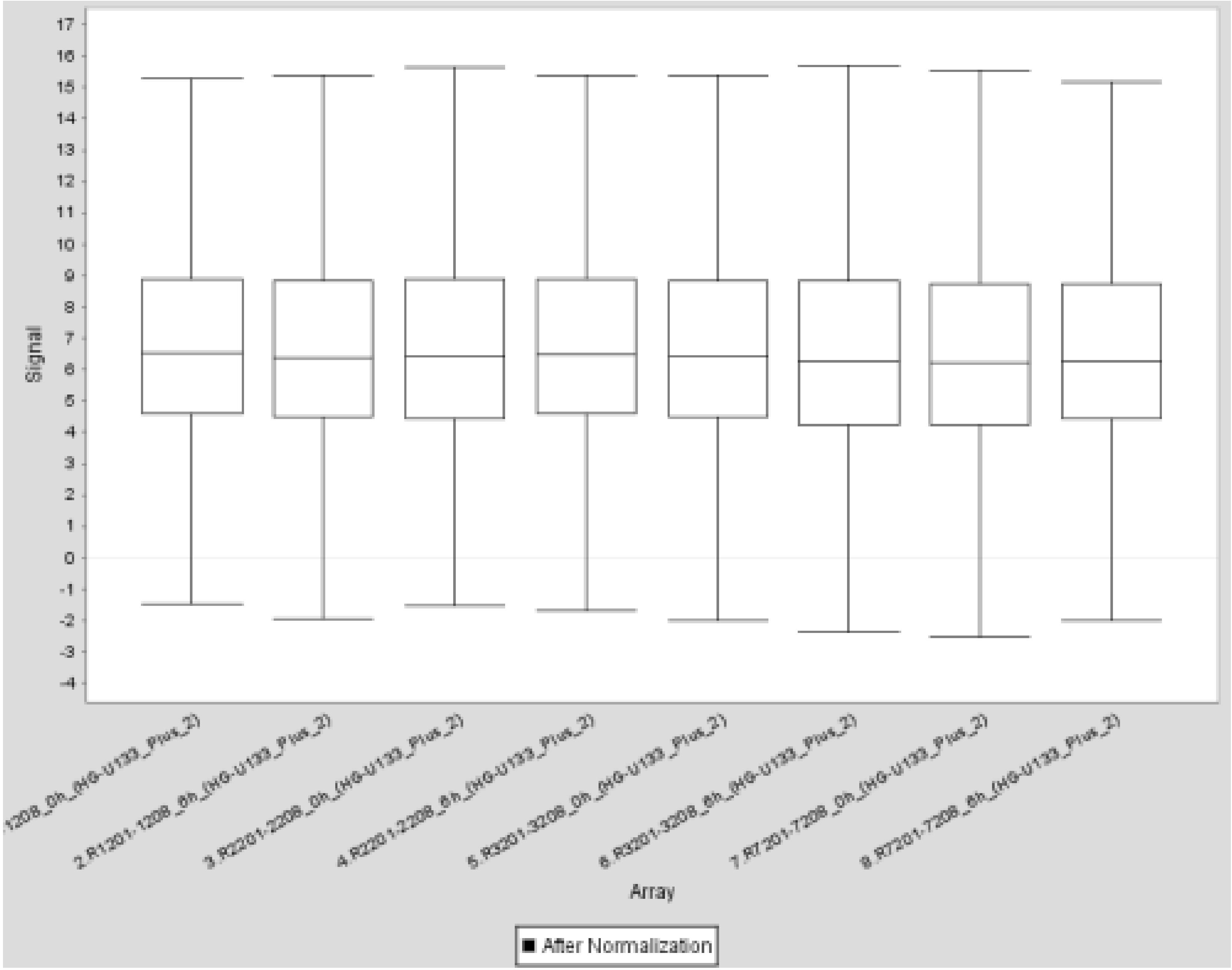

Supplementary Figure 1 (B)

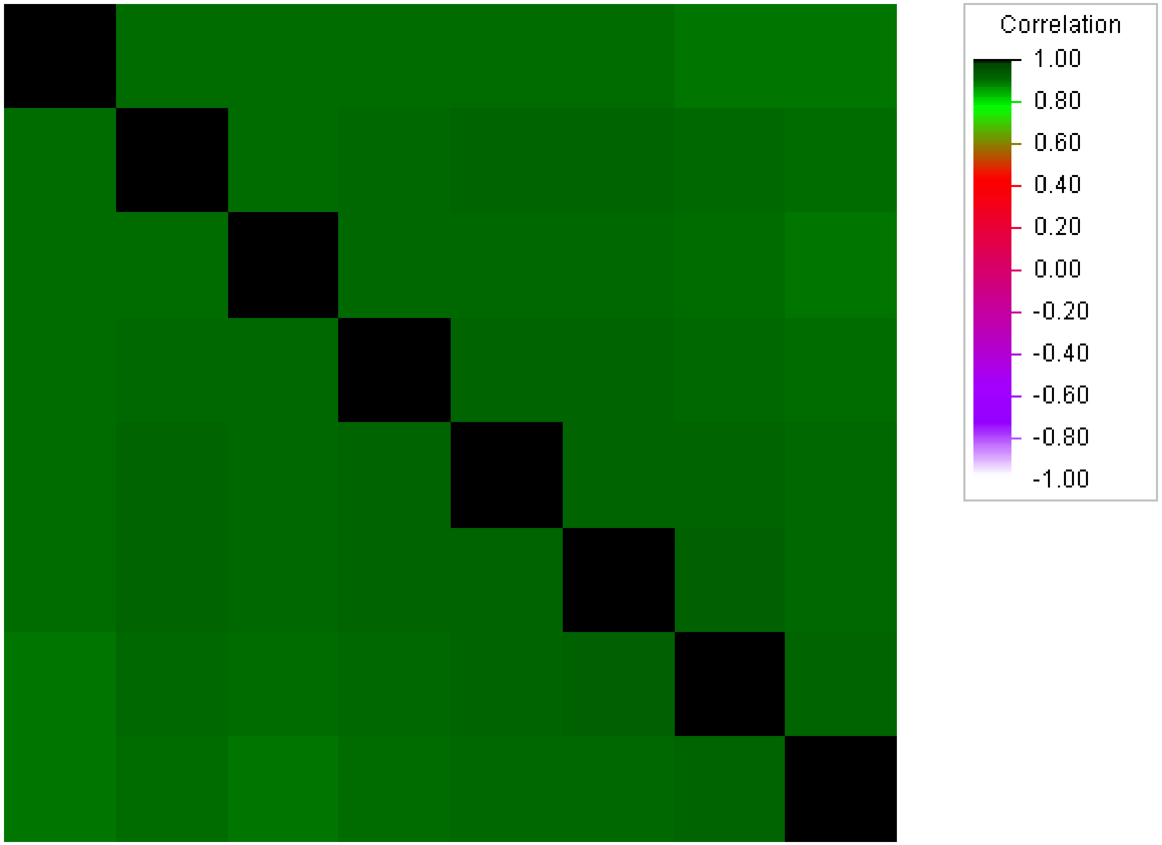

Supplementary Figure 1 (C)

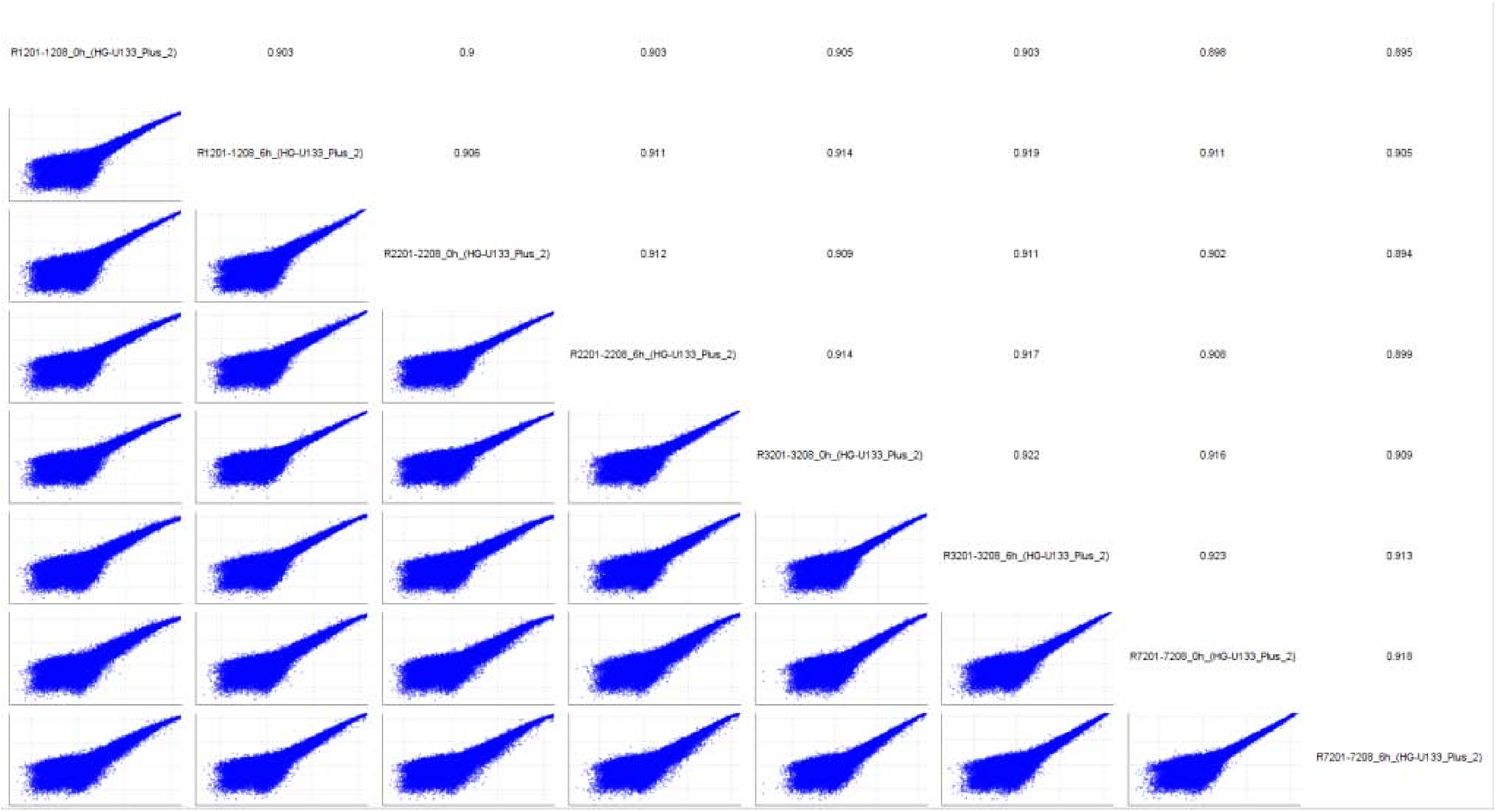

Supplement: Supplementary Information [file srep17784-s1.pdf]
